# Buffer Exchange, a Simple Option That Significantly Increases the Chances of Isolation of Adenoviruses from Blood and Other Clinical Specimens

**DOI:** 10.1128/spectrum.00454-23

**Published:** 2023-05-16

**Authors:** Adriana E. Kajon

**Affiliations:** a Lovelace Biomedical Research Institute, Albuquerque, New Mexico, USA; University of Arizona

**Keywords:** adenovirus, isolation, blood specimens, EDTA

## LETTER

Through precious opportunities afforded to my laboratory over the last 8 years by collaborative research projects funded by the National Institute of Allergy and Infectious Diseases to process blood specimens (whole blood, plasma, occasionally serum) to attempt the isolation and characterization of human adenoviruses (HAdVs) infecting pediatric transplant recipients, we have repeatedly been challenged by the high toxicity of these samples to cells in culture. Here, we share important lessons learned from troubleshooting this critical obstacle. After a couple of rounds of loss of all inoculated cultures without being able to determine why whole blood, and plasma specimens in particular (sometimes also urine and stool specimens), lifted cell monolayers on contact, we hypothesized two candidate sources of cell toxicity: patient medications and anticoagulants in the collection tube. We started thinking about the need to somehow clean up these irreplaceable samples and also researching the specifications of blood collection tubes from various manufacturers. We rapidly learned that lavender top tubes (of any brand) that are regularly used for hematological testing provide a final concentration of ~1.8 mg K_2_EDTA per milliliter of blood as an anticoagulant (1). This concentration is 10 times higher than that of Versene, the EDTA solution widely used as a nonenzymatic cell dissociation reagent and formulated as 0.2 g/L EDTA(Na_4_) in phosphate-buffered saline (PBS), explaining why plasma and EDTA-whole blood specimens were poorly—or not at all—tolerated by A549 cell monolayers in shell vials or culture tubes. Interestingly, we could not find any reports of similar observations in the literature, only succinct descriptions of sample dilution before inoculation with no rationale or explanation provided.

Because many of our research projects require cleanups or buffer exchanges for virus stock preparation, we decided to try 4-mL centrifugal filter devices with a 100-kDa molecular weight cutoff that we had in the lab (Millipore-Sigma Amicon Ultra-4; UFC810024). We implemented a protocol where we mixed one volume of plasma (or whole blood) with three volumes of PBS and followed the manufacturer’s instructions for centrifugation and sample recovery from the filter. This simple procedure has made a huge difference in our ability to maintain original specimens in culture long enough to monitor the development of a cytopathic effect. We have never had abundant specimens to conduct a formal comparison of the performance in culture of treated (buffer-exchanged) versus untreated samples to obtain a large data set and write a methods paper. We can, however, definitively share that this strategy has worked well in our hands and helped keep infected cell cultures viable for several days after inoculation, increasing our ability to recover infectious HAdVs of various species and types from many blood specimens within one or two passages.

The recent opportunity to handle a large number of pediatric blood specimens (obtained under informed consent) testing positive for HAdV by quantitative PCR (qPCR) with known viral loads allowed us to make valuable preliminary observations of correlations between the load of detectable viral DNA, molecular diagnostic viability, and culturability of the virus from clinical specimens (see representative data in Table 1). This important topic warrants further investigation.

**TABLE 1:** Viral load and culturability of HAdVs from a representative set of qPCR-positive pediatric blood specimens obtained from 5 deidentified sources (sites 1 to 5) and recently processed in the Kajon lab for virus isolation after buffer exchange^*a*^

Specimen type	Site	Viral load by qPCR (log_10_)	Original vol processed	Culturable in A549^*b*^	No. of passages for isolation	Isolated type	Hexon PCR seq^*d*^
Plasma/EDTA	1	<2	200 μL	No			B34
Plasma/EDTA	1	<2	200 μL	No			A31
Plasma/EDTA	2	3.64	200 μL	No			A12
Plasma/EDTA	3	4	200 μL	No			F41
Plasma/EDTA	2	4.17	200 μL	No			C1
Plasma/EDTA	4	4.57	200 μL	No			C5
Plasma/EDTA	4	4.88	200 μL	No			B35
Plasma/EDTA	2	5	200 μL	No			A12
Plasma/EDTA	1	5.69	200 μL	Yes	2	C5	C5
Plasma/EDTA	1	5.70	200 μL	Yes	2	C5	C5
Plasma/EDTA	2	6.19	200 μL	No			C1
Plasma/EDTA	2	6.24	200 μL	No			C2
Plasma/EDTA	2	6.35	200 μL	Yes	2	B14	B14
Plasma/EDTA	2	6.94	200 μL	Yes	2	B14	B14
Whole blood	2	7.77	500 μL	Yes	1	C1	C1
Whole blood	2	7.88	500 μL	Yes	1	C1	C1
Whole blood	5	8	1 mL	Yes	2	C108	C1
Plasma/EDTA	5	8	300 μL	Yes	2	C108	C1
Blood clot^*c*^	5			Yes	2	C108	C1
Plasma/EDTA	2	9.53	200 μL	Yes	1	C5	C5
Plasma/EDTA	2	9.71	200 μL	Yes	1	C5	C5

a Samples from which we obtained an isolate are highlighted with shading.

b Within 5 serial passages.

c This sample was not processed for buffer exchange; a homogenate was generated and clarified by centrifugation before inoculation of the cells into culture. The corresponding serum sample had a log_10_ viral load of 6.92.

d All samples were typeable by hexon PCR and amplicon Sanger sequencing following the protocol originally developed by Okada et al. (2) and previously implemented in some of our surveillance studies conducted in collaboration with the New York State Department of Health (3, 4).

Based on what we have learned from this experience, we recommend that a buffer exchange step be considered during specimen processing to improve the chances of isolation of adenoviruses from blood. Importantly, the principle of the method allows for sample concentration, which is highly desirable for specimens with a low viral load. The applicability of buffer exchange to facilitate the recovery of viruses of other families from blood should be explored.
